# Nanoemulsion Hydrogel Delivery System of *Hypericum perforatum* L.: In Silico Design, In Vitro Antimicrobial–Toxicological Profiling, and In Vivo Wound-Healing Evaluation

**DOI:** 10.3390/gels11060431

**Published:** 2025-06-03

**Authors:** Ahmet Arif Kurt, Bashar Ibrahim, Harun Çınar, Ayşe Nilhan Atsü, Ertuğrul Osman Bursalıoğlu, İsmail Bayır, Özlem Özmen, İsmail Aslan

**Affiliations:** 1 Faculty of Pharmacy, Department of Pharmaceutical Technology, Süleyman Demirel University, Isparta 32000, Türkiye; 2 Faculty of Pharmacy, Department of Pharmaceutical Microbiology, Süleyman Demire University, Isparta 32000, Türkiye; basharibrahim@sdu.edu.tr; 3 Faculty of Veterinary Medicine, Department of Veterinary Surgery, Burdur Mehmet Akif Ersoy University, Burdur 15100, Türkiye; hcinar@mehmetakif.edu.tr; 4 Vocational School, Istanbul Kent University, İstanbul 34406, Türkiye; aysenilhan.atsu@kent.edu.tr; 5 Vocational School of Health Services, Sinop University, Sinop 57000, Türkiye; ebursalioglu@sinop.edu.tr; 6 Vocational School, Sakarya University, Sakarya 54100, Türkiye; 7 Vocational School, Erzincan Binali Yıldırım University, Erzincan 24100, Türkiye; ibayir@erzincan.edu.tr; 8 Faculty of Veterinary Medicine, Department of Pathology, Burdur Mehmet Akif Ersoy University, Burdur 15100, Türkiye; ozlemoz@mehmetakif.edu.tr; 9 Hamidiye Faculty of Pharmacy, Department of Pharmaceutical Technology, University of Health Sciences, İstanbul 34668, Türkiye; ismail.aslan@sbu.edu.tr; 10 Faculty of Pharmacy, Istanbul Kent University, İstanbul 34406, Türkiye

**Keywords:** hydrogel, nanoemulsion, wound healing, HET-CAM, drug delivery systems, antimicrobial activity, *Hypericum perforatum* L., in silico modeling, Cremophor RH40, antioxidant activity

## Abstract

*Hypericum perforatum* L. (H.P.), a plant renowned for its wound-healing properties, was investigated for antioxidant/antimicrobial efficacy, toxicological safety, and in vivo wound-healing effects in this research to develop and characterize novel nanoemulsion hydrogel (NG) formulations. NG were prepared via emulsion diffusion–solvent evaporation and polymer hydration using Cremophor RH40 and Ultrez 21/30. A D-optimal design optimized oil/surfactant ratios, considering particle size, PDI, and drug loading. Antioxidant activity was tested via DPPH, ABTS^+^, and FRAP. Toxicological assessment followed HET-CAM (ICH-endorsed) and ICCVAM guidelines. The optimized NG-2 (NE-HPM-10 + U30 0.5%) demonstrated stable and pseudoplastic flow, with a particle size of 174.8 nm, PDI of 0.274, zeta potential of −23.3 mV, and 99.83% drug loading. Release followed the Korsmeyer–Peppas model. H.P. macerates/NEs showed potent antioxidant activity (DPPH IC_50_: 28.4 µg/mL; FRAP: 1.8 mmol, Fe^2+^/g: 0.3703 ± 0.041 mM TE/g). Antimicrobial effects against methicillin-resistant *S. aureus* (MIC: 12.5 µg/mL) and *E. coli* (MIC: 25 µg/mL) were significant. Stability studies showed no degradation. HET-CAM tests confirmed biocompatibility. Histopathology revealed accelerated re-epithelialization/collagen synthesis, with upregulated TGF-β1. The NG-2 formulation demonstrated robust antioxidant, antimicrobial, and wound-healing efficacy. Enhanced antibacterial activity and biocompatibility highlight its therapeutic potential. Clinical/pathological evaluations validated tissue regeneration without adverse effects, positioning H.P.-based nanoemulsions as promising for advanced wound care.

## 1. Introduction

In the contemporary era, characterized by the advancement of nanotechnology and polymer technology, the development of nano-sized drug carrier systems with physical and chemical properties has become a pivotal research area and focus in the design and production of new pharmaceutical products. Polymers that are sensitive to environmental stimuli, also referred to as ‘smart gels’ in the literature, offer a wide range of applications, especially in the fields of medicine, pharmaceutical sciences, and biotechnology [1,2,3]. Hydrogels containing nanoemulsions have led to an increasing intensification of scientific research in this field due to their potential applications in optical, electrical, chemical, biological, and medical devices. It is anticipated that these advancements will serve as a significant catalyst for ongoing research endeavors in this domain.

Wound healing is a complex and dynamic process characterized by a series of cellular and molecular events that work to restore tissue integrity [4]. This process consists of three main phases: inflammation, proliferation, and remodeling, each of which is influenced by various intrinsic and extrinsic factors. A major challenge in wound healing is the risk of infection, which can delay recovery, contribute to the formation of chronic wounds, and significantly increase treatment costs. Given these complications, there is a growing demand for innovative therapeutic approaches that can accelerate wound healing while simultaneously preventing infections [5].

Herbal medicines, which have been integral to traditional medical practices for centuries, have gained significant attention for their wound healing [6,7] and antimicrobial characteristics [8,9,10,11]. Among these, *Hypericum perforatum* L. (St. John’s wort) and *Olea europaea* (olive oil) have been widely recognized for their therapeutic potential, attributed to their bioactive compounds [12]. St. John’s wort is particularly valued for its anti-inflammatory and wound-healing effects, which are primarily linked to its key constituents, hypericin and hyperforin [13]. Similarly, olive oil has been associated with skin health benefits, largely due to its antioxidant and anti-inflammatory properties [14,15]. However, in the present study, olive oil did not exhibit antimicrobial activity, which raises questions about the factors influencing its efficacy.

One possible explanation for this discrepancy could be insufficient concentrations of bioactive components (e.g., polyphenols) in the tested oil. Alternatively, variations in experimental conditions or differences in olive oil composition across studies may account for the observed lack of activity. This suggests that the antimicrobial efficacy of olive oil may be dose-dependent and influenced by factors such as the specific microbial strain and interactions within the formulation.

Nanotechnology has revolutionized drug delivery systems, with nanoemulsions emerging as a particularly promising strategy for enhancing the efficacy and bioavailability of herbal actives [16]. The advantages of the nanoemulsion stem from their small particle size, high surface area, and structural stability, which collectively enable targeted delivery, controlled release, and improved solubility of bioactive compounds. These properties are especially beneficial in wound-healing applications, where sustained and localized release of therapeutic agents can optimize treatment outcomes.

Simultaneously, hydrogel formulations have gained attention for their ability to support wound healing by providing a moist wound environment, which is crucial for cell migration, proliferation, and tissue regeneration [17]. Additionally, their biocompatibility and structural integrity make them ideal carriers for nanoemulsion-based delivery systems, further enhancing their therapeutic potential.

The development of nanoemulsions has been a particular area of focus in this field. These systems exhibit noteworthy antimicrobial properties, attributable to their distinctive electrical, chemical, mechanical, and optical characteristics, along with their substantial surface area-to-volume ratio and minuscule size [18]. These attributes position nanoemulsions as a promising platform for both antimicrobial applications and the diagnosis and treatment of diseases [19]. In the domains of drug targeting, antimicrobial therapies, and cancer therapy, research with nanoemulsions has yielded significant scientific and clinical advances. In contrast, hydrogels are three-dimensional, cross-linked polymer networks that exhibit the ability to undergo swelling without dissolution in water and are sensitive to various environmental stimuli, including pH, temperature, ionic force, electric field, and the presence of enzymes. Their biocompatibility, demonstrated by their structural similarity to living tissues in the swollen state, and their soft and elastic properties, render them highly attractive materials for biomedical applications [20].

Biological methods employed in the synthesis of nanoemulsions present an environmentally friendly and sustainable alternative to conventional chemical and physical synthesis techniques. This biosynthesis strategy involves the utilization of biological resources, including microorganisms, algae, enzymes, plants and plant extracts. These sources possess a variety of secondary metabolites with high reducing potential, thus enabling their function as both reducing and stabilizing agents in the synthesis process. In recent years, there has been a notable increase in research focusing on plant-based systems for nanoemulsion synthesis, with numerous plant species and their extracts being studied for this purpose [21].

*Hypericum perforatum* L., naturally distributed in Türkiye, is employed in traditional medicine for a variety of therapeutic purposes and is known by several vernacular names, including “Sarı Kantaron”, “Binbir Delik Otu”, “Yara Otu”, “Kanotu”, “Mayasıl otu”, and “Kuzukıran”. This diverse nomenclature reflects regional variations in usage and knowledge [22]. *Hypericum perforatum* L. is commonly observed in roadside habitats, arid landscapes, and calcareous soils. Its geographical distribution extends beyond Türkiye, with documented occurrences in regions exhibiting diverse climatic conditions, encompassing the Asian continent, Iran, Cyprus, and the Mediterranean basin [22,23]. Phytochemical characterization of the plant reveals the presence of a range of bioactive compounds, including hypericin and pseudohypericin (0.05–0.3%), hyperforin and adhyperforin (0.9–5.0%), and various flavonoids (approximately 4%). These constituents have been identified as the primary pharmacological agents responsible for the plant’s therapeutic bioactivities [24,25]. *Hypericum perforatum* L., while the most prevalent species within the Hypericum genus, is also considered an endemic species in certain regions. Preclinical studies have demonstrated that topical application of *Hypericum perforatum* macerate promotes the healing of cutaneous injuries, including sunburns, minor abrasions, and superficial wounds. Consequently, it exhibits potential as a therapeutic intervention for various dermatological conditions [26].

Antibiotics are agents commonly used in the treatment of infectious diseases. They provide treatment by inhibiting bacterial cell growth and proliferation; this effect is achieved through inhibition of bacterial proliferation or inhibition of cell wall synthesis. The integration of nanotechnology into medical applications has spawned the burgeoning field of ‘nanomedicine’. Surface functionalization of nanoemulsions (NEs) can augment their antimicrobial and antineoplastic properties, while concurrently mitigating potential cytotoxic effects [27]. Therefore, single-step synthesis of nanoparticles presents a promising avenue for enhancing biocompatibility and facilitating their incorporation into future pharmaceutical formulations.

Given these advancements, the integration of nanoemulsion and hydrogel technologies presents a synergistic approach for wound care and drug delivery, combining enhanced bioavailability, sustained release, and an optimal wound-healing environment. However, further research is necessary to explore the long-term stability, biocompatibility, and clinical efficacy of these formulations to fully harness their potential in regenerative medicine and targeted therapy.

In this research, a novel hydrogel–nanoemulsion system incorporating macerates of *Hypericum perforatum* L., and Olea europaea was designed. The objectives encompassed evaluating the in vitro antioxidant activity, antimicrobial efficacy, and in vivo wound-healing capacity of this formulation, and leveraging synergistic phytochemical and nanotechnological approaches to advance topical therapeutic interventions.

## 2. Results and Discussion

### 2.1. In Silico Modeling and Optimization

The software generated 11 distinct formulation outputs based on the input parameters. The in silico design space incorporated *Hypericum perforatum* macerate (6–9%), surfactant (Cremophor RH40 BASF, Ludwigshafen, Germany, 5–8%), and ultrapure water, varying the ratios of these components to predict formulation properties. The experimental data derived from the prepared formulations, combined with the established parameters, were used to construct three-dimensional (3D) response surface plots (Table 1).

**Figure 1:**
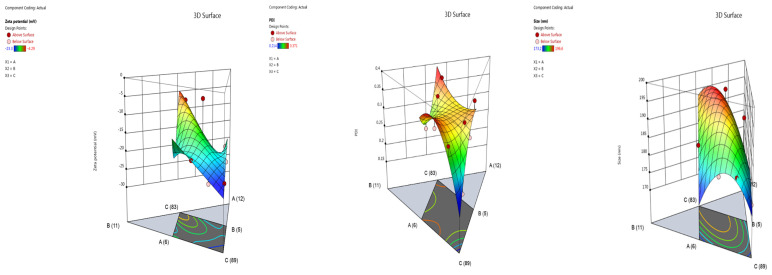
Three-dimensional (3 D) response surface contour plots were generated to visualize the relationships between the independent variables (A-*Hypericum perforatum* L. macerate and B-surfactant concentration (Cremophor RH-40)) and the dependent variables (particle size, particle size distribution, and zeta potential) based on the quadratic calibration model employed in the in-silico modeling (DoE-Design Expert 13).

Following a detailed analysis of the 3D response surfaces and the influence of each independent variable on the dependent variables, nanoemulsion formulation NE-HPM-10 was identified as the optimal formulation (Figure 1).

The integration of nanoemulsions within a hydrogel matrix represents a synergistic approach with significant potential for advancing both wound healing and antimicrobial therapeutic strategies. In silico studies on nanoemulsion selection revealed 11 distinct nanoemulsion formulations, each characterized by specific concentration ratios of *H. perforatum* L. macerate (HPM), Cremophor RH40 (C-RH40), and ultrapure water (U.W.) components. These concentration ratios are variable and correspond to the formulation codes. Figure 1 presents three-dimensional (3D) response surface contour plots, which have been created to visualize the relationships between independent variables (*H. perforatum* L. macerate and surfactant concentration [Cremophor RH-40]) and dependent variables. These plots provide a visual representation of how the independent variables affect the dependent variables. The in silico modeling program DoE Expert suggested 11 formulations in different combinations.

### 2.2. Particle Size, Polydispersity Index, Zeta Potential, and Encapsulation Efficiency

Particle size, polydispersity index (PDI), zeta potential, and encapsulation efficiency are critical parameters in determining the optimal nanoemulsion formulation. The selected nanoemulsion formulations loaded with *H. perforatum* L. macerate (HPM) are presented in Table 2 (*n* = 3).

The quantification of constituents in the HPM content was performed following the methodology of previous studies and in compliance with the ICH Q2 (R1) guideline [28,29]. NE-HPM-10 was identified as the formulation with the highest encapsulation efficiency among HPM-loaded nanoemulsions, achieving a value of 99.83 ± 3.09%. The particle size, polydispersity index, and zeta potential of this formulation were determined as 174.8 ± 1.436 nm, 0.274 ± 0.023, and −23.3 ± 0.2 mV, respectively. These findings indicate that NE-HPM-10 exhibited more favorable characteristics compared to other formulations and is expected to maintain its stability throughout its shelf life.

As demonstrated in Table 2, a comprehensive evaluation of nanoemulsion formulations loaded with *H. perforatum* L. macerate (HPM) was conducted, with parameters including particle size, polydispersity index (PDI), zeta potential, and encapsulation efficiency. The findings reveal that a small particle size, PDI values ranging from 0.2 to 0.5, zeta potential values of approximately −25 mV or +25 mV in accordance with DLVO theory, and encapsulation efficiency close to 100% for lipophilic actives are crucial parameters in formulation optimization [30,31,32]. The results demonstrate that the encapsulation efficiency is optimal in the NE-HPM-10 formulation, with a value of 99.83 ± 3.09%, making it the most suitable formulation among the 11 formulations presented. In this research, we successfully fabricated stable nanoemulsions with a mean particle size of 174.8 ± 1.436 nm and a zeta potential of −23.3 ± 0.2 mV, demonstrating a high encapsulation efficiency of 99.83 ± 3.09% for the optimum nanoemulsion formulation. The successful encapsulation of *H. perforatum* L. macerate within the nanoemulsion system further validates the suitability of nanoemulsions as effective drug delivery vehicles for hydrophobic bioactive compounds, ensuring their stability and sustained release at the target site.

### 2.3. SEM Images

**Figure 2:**
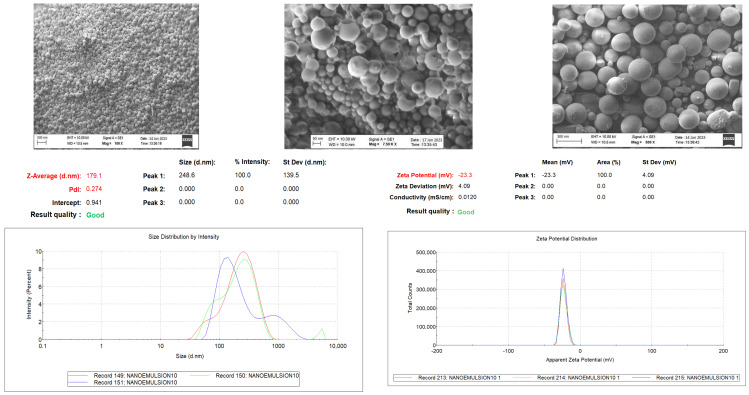
SEM images, particle size distribution, and zeta potential graphs of NE-HPM10 (*n* = 3).

Scanning electron microscopy (SEM) (Zeiss EVO 40, Jena, Germany) was performed to characterize the morphology of the optimal nanoemulsion formulation, NE-HPM-10. The resulting images revealed that the nanoparticles exhibited a generally uniform, spherical morphology. Furthermore, the SEM micrographs indicated that the particle size distribution within NE-HPM-10 was largely homogeneous, with the majority of particles exhibiting dimensions below 200 nm, confirming their nanoscale nature (Figure 2).

SEM images were obtained to ascertain the shape of the nanoemulsions in the optimum formulation, NE-HPM-10, and it was established that they generally have a uniform spherical shape. SEM images of NE-HPM-10 show that the particle sizes are mostly homogeneous and the particle size is below 200 nm at the nanoscale.

Following the optimization of the nanoemulsion formulation, an examination was conducted of the morphology of the formulation by means of SEM. It was established that the NE-HPM-10 formulation, which exhibited the highest encapsulation efficiency among the nanoemulsions that were subjected to testing, also demonstrated the presence of desirable physical properties with regard to particle size, morphology, and homogeneity. Electron microscopy images were found to be in agreement with the data obtained from the DLS device, thus indicating the integrity of the surfaces and the stable placement of the nanoemulsions (see Figure 2).

### 2.4. Rheological Analysis

**Figure 3:**
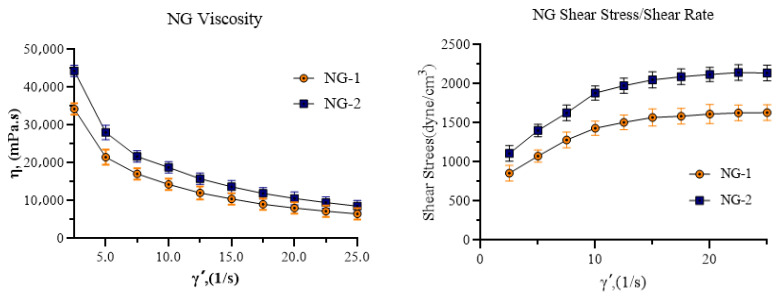
Viscosity, shear stress, and shear rate graphs of NG-1 (containing Ultrez 21) and NG-2 (containing Ultrez 30).

A comprehensive rheological analysis was performed on carbomer-based hydrogel formulations, specifically NG1 (U21-containing hydrogel) and NG2 (U30-containing hydrogel). This analysis included the measurement of viscosity and shear stress responses, as well as an investigation of the changes in viscosity and shear stress of the gelling agents U-21 and U-30 across different pH levels. The rheological behavior of the hydrogel formulations, exhibiting non-Newtonian flow, is illustrated in Figure 3.

The rheological assessment of NG-1 (Ultrez 21) and NG-2 (Ultrez 30) nanoemulsion hydrogels confirmed their non-Newtonian, shear-thinning profiles, supporting their applicability for topical drug delivery. Specifically, NG-2 exhibited higher viscosity values across a range of shear rates compared to NG-1, suggesting greater mechanical stability and resistance to deformation under stress. This increased viscosity may contribute to the improved mucoadhesive and occlusive properties of NG-2, providing a more favorable microenvironment for wound healing by maintaining moisture and facilitating controlled drug release. It is also noteworthy that environmental stimuli such as pH and temperature may differentially affect the cross-linking density and swelling behavior of U21 and U30-based hydrogels, potentially altering their rheological profiles in situ. Given that wound environments typically exhibit dynamic changes in pH and hydration levels, a more comprehensive investigation of the rheological behavior of these hydrogels under physiologically relevant conditions would further substantiate their clinical applicability.

### 2.5. Release Kinetic

**Figure 4:**
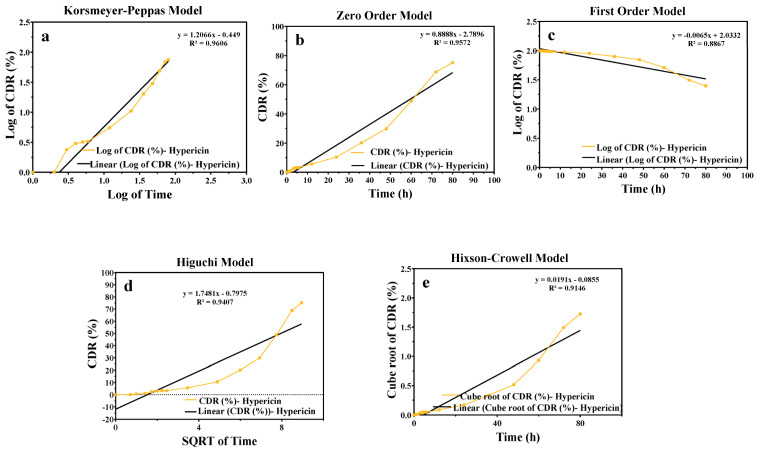
Release kinetic graphs of NG-2 formulation: (**a**) Korsmeyer–Peppas; (**b**) zero order; (**c**) first order; (**d**) Higuchi; (**e**) Hixson–Crowell kinetic models. CDR: cumulative drug release quantity, SQRTT: square root of time.

The release kinetics of the active component from the NG-2 hydrogel formulation were evaluated using various mathematical models, including zero-order, first-order, Higuchi, Korsmeyer Peppas, and Hixson Crowell models, in accordance with previously established methodologies for nanoemulsion systems [29]. The models were generated based on the data obtained from drug release studies, as presented in Table 3 and Figure 4.

The drug release profiles presented in Figure 4 effectively demonstrate the release behavior of the active ingredient from the NG-2 hydrogel formulation, which can be effectively modeled using the Korsmeyer–Peppas equation. This equation was selected as the most appropriate release model. The NG-2 hydrogel formulation is of the Non-Newtonian type, making it suitable for topical applications.

In order to better assess the potential of the NG-2 hydrogel formulation, it is essential to compare its performance with existing wound-healing products. Conventional hydrogel dressings, although widely used, often suffer from limited drug-loading capacity and uncontrolled drug release profiles, resulting in suboptimal therapeutic outcomes. Commercial antibiotic creams, on the other hand, provide rapid antibacterial action but typically lack sustained release behavior and moisture retention, which are critical for effective wound healing. In contrast, the NG-2 hydrogel demonstrated both a sustained release of the active compound and favorable physicochemical properties, including prolonged moisture maintenance and enhanced biocompatibility, indicating its potential as an advanced alternative to conventional treatments.

### 2.6. In Vitro Release Profile

**Figure 5:**
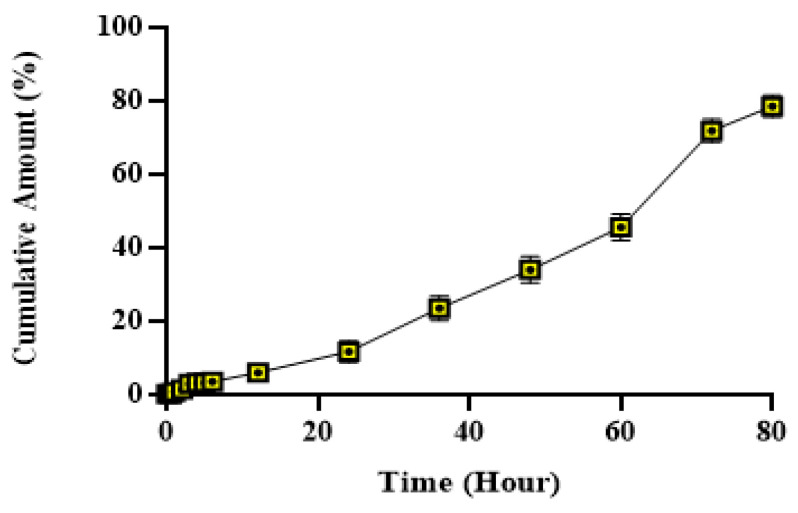
Graph of % cumulative hypericin content versus time.

The in vitro release of hypericin was assessed using the dialysis bag method with quantification by HPLC at 270 nm. The cumulative percentage of hypericin released over time, as determined from samples collected at 0.5, 1, 2, 3, 4, 5, 6, 12, 24, 36, 60, 72, and 80 h, is depicted in Figure 5. The release profile indicates a sustained and controlled release pattern for up to 10 h. By the end of the 80 h period, a total hypericin release of 75.12 ± 3.90% was observed.

Although both the nanoemulsion (NE-HPM-10) and nanoemulsion–hydrogel (NG-2) formulations exhibited sustained release profiles, the integration of the nanoemulsion into a carbomer-based hydrogel matrix in the NG-2 formulation significantly prolonged the release of hypericin up to 80 h, as demonstrated by in vitro dialysis studies. This extended release profile, best described by the Korsmeyer–Peppas model, indicates that the hydrogel network contributes to diffusion-controlled kinetics. The reason for including only the NG-2 formulation in the in vivo evaluation was based on the aim of reducing the number of animal groups in accordance with the 3Rs principle (Replacement, Reduction, Refinement), alongside NG-2’s superior clinical relevance as a topical delivery system. Hydrogels minimize the initial burst release effect frequently observed with nanoemulsions and create a moist wound environment, which is critical for tissue regeneration. Additionally, NG-2 provides improved rheological properties and mechanical stability due to the presence of Ultrez 30 polymer, ensuring longer retention at the wound site and enabling controlled drug delivery. The sustained and controlled release profile of the NG-2 formulation demonstrates the potential for effective drug delivery and therapeutic efficacy.

### 2.7. Stability Study Results of NG-2 Nanoemulsion Hydrogel Formulation

The stability of the NG-2 nanoemulsion hydrogel formulation was evaluated over a 4-month period under accelerated conditions and results were given in Table 4, following International Council for Harmonisation (ICH) guidelines.

The results obtained from measurements conducted at regular intervals over a period of four months at a temperature of +4 °C indicate that nanoemulsion formulations were able to preserve the size, polydispersity index, zeta potential, encapsulation efficiency, viscosity, and pH values. Initially, the size, zeta potential, and polydispersity index for the NE-HPM-10 nanoemulsion formulation were 173.19 ± 4.3 (d.nm), −24.2 ± 3.7 (mV), and 0.214 ± 0.092, respectively. The encapsulation efficiency and pH values at +4 °C were 99.09 ± 0.92% and 7.322 ± 0.04, respectively. According to the results of the stability studies carried out at +25 °C, the size values increased slightly more than +4 °C at the end of four months and were measured as 221.457 ± 3.900 (d.nm). The PDI value was measured as 0.208 ± 0.022 in the third month and 0.324 ± 0.05 in the fourth month, indicating that the values of other parameters preserved their physicochemical properties. In regard to the test results simulating refrigerator and humid climatic conditions, we found that the changes in dimensions were below 300 (d.nm) at the end of the fourth month. The PDI value was determined to be 0.284 ± 0.022 in the third month and 0.355 ± 0.05 in the fourth month, indicating a shift towards a more heterogeneous size distribution over time. The analysis concluded that 4 °C storage conditions were more suitable than 25 °C and 40 °C.

### 2.8. In Vitro Antimicrobial Efficacy

This study evaluated the antimicrobial efficacy of various agents, including olive oil (O-O), *H. perforatum* L. macerate (HPM), nanoemulsion *H. perforatum* L. macerate-10 (NE-HPM-10), CP1 (a commercial product comprising a topical herbal gel formulation containing *Hypericum perforatum* extract, *Melia azadirachta* seed oil, and *Olea europaea* oil), CP2 (a commercial product comprising an ointment containing 10 mg/g of standardized *Centella asiatica* extract, formulated with terpene-free essential oils and emulsifying excipients), and CP3 (commercial product comprising a petrolatum-based topical antibacterial ointment composed of 30 mg/g oxytetracycline hydrochloride and 10,000 IU/g polymyxin B sulfate). Their antimicrobial activity was tested against six bacterial species: *Escherichia coli*, *Pseudomonas aeruginosa*, *Enterococcus faecalis*, *Staphylococcus aureus*, methicillin-resistant *Staphylococcus aureus (MRSA)*, and *Bacillus cereus*. The minimum inhibitory concentration (MIC) and minimum bactericidal concentration (MBC) values were determined to assess their effectiveness.

The results revealed differential antimicrobial activity across the tested microorganisms. Notably, CP3 exhibited the highest antimicrobial activity against Gram-negative and Gram-positive bacteria, as indicated by the lowest MIC and MBC values. NE-HPM-10 demonstrated superior antimicrobial activity compared to olive oil and HPM, supporting the hypothesis that nanoemulsion formulations enhance antimicrobial efficacy.

These findings suggest that nanoemulsion-based formulations, such as NE-HPM-10, possess broad-spectrum antimicrobial potential and may serve as promising candidates for infection prevention in wound healing applications. The robust performance of CP3, a commercial antibiotic, across all tested bacteria aligns with its expected efficacy as a positive control, thereby reinforcing the methodological rigor of this study.

**Figure 6:**
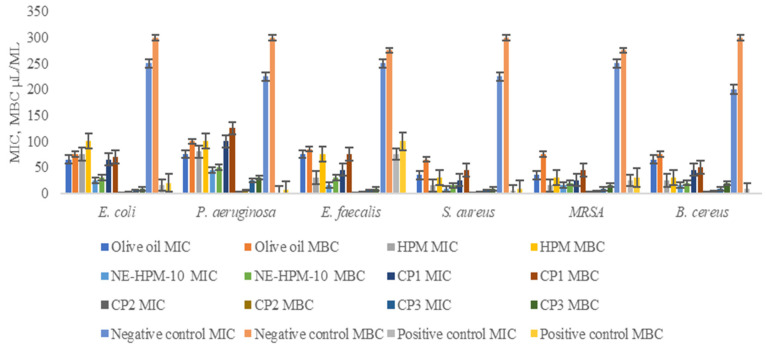
Minimum inhibitory (MIC) and minimum bactericidal/fungicidal (MBC/MFC) concentrations of different antimicrobial agents. Groups: 1–olive oil, 2—HPM (*H. perforatum* L. macerate), 3—NE-HPM-10, 4—CP1, 5–6—CP2, 7—CP3, 8—negative control (S.F.), 9—positive control (Sefalosporin).

Collectively, the results highlight the potential of nanoemulsion formulations (NE-HPM-10) as an alternative or complementary approach to conventional synthetic antibiotics, warranting further investigation (see Figure 6).

The in vitro antimicrobial assays revealed that the HPM-loaded nanoemulsions exhibited varying degrees of inhibitory activity against a panel of clinically relevant microorganisms. Notably, the optimized formulation NE-HPM-10 demonstrated significant antimicrobial potential, particularly against methicillin-resistant *Staphylococcus aureus* (MRSA), as evidenced by its low MIC and MBC values. This suggests that nanoemulsification enhances the antimicrobial activity of HPM, potentially overcoming inherent limitations associated with its direct application. Conversely, *H. perforatum* macerate alone exhibited comparatively lower antimicrobial activity against certain microorganisms, further highlighting the importance of formulation design and compositional optimization in maximizing therapeutic efficacy. The data presented in Figure 6 summarize the MIC and MBC/MFC values for the different antimicrobial agents tested and highlight the superior performance of the nanoemulsion formulation NE-HPM-10 on the bacterial species evaluated.

### 2.9. Antioxidant Activity

**Figure 7:**
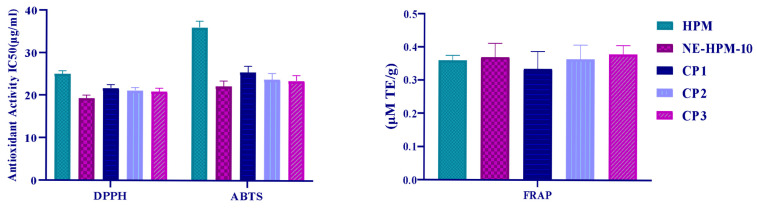
DPPH, ABTS, and FRAP antioxidant activity results.

DPPH, ABTS, and FRAP antioxidant activity results of NG-2 formulation showed that it was effective and were given in Figure 7. DPPH (2,2-diphenyl-1-picrylhydrazyl) radicals at an IC50 concentration: HPM, NE-HPM-10, CP1, CP2, and CP3. The IC50 (μg/mL) values for HPM, NE-HPM-10, CP1, CP2, and CP3 were found to be 25.030 ± 0.760 μg/mL, 19.308 ± 0.714 μg/mL, 21.708 ± 0.813 μg/mL, 21.134 ± 0.708 μg/mL, and 20.922 ± 0.0.803 μg/mL, respectively. The developed formulations demonstrate comparable levels of antioxidant activity.

The ability to inhibit ABTS (Diammonium 2,2′-azino-bis (3-ethylbenzothiazoline-6-sulfonate)) radicals was investigated, with the IC50 (μg/mL) values for HPM, NE-HPM-10, CP1, CP2, and CP3 being 36.024 ± 1.504 μg/mL, 22.125 ± 1.289 μg/mL, 25.505 ± 1.433 μg/mL, 23.819 ± 1.487 μg/mL, and 23.519 ± 1.287 μg/mL, respectively.

The ferric ion reducing antioxidant powers of the HPM, NE-HPM-10, CP1, CP2, and CP3 products were 0.3598 ± 0.016 mM TE/g, 0.3703 ± 0.041 mM TE/g, 0.3352 ± 0.052 mM TE/g, 0.3646 ± 0.043 mM TE/g, and 0.3792 ± 0.027 mM TE/g, respectively.

### 2.10. Hen’s Egg Test Chorioallantoic Membrane (HET-CAM) Assay Results

**Figure 8:**
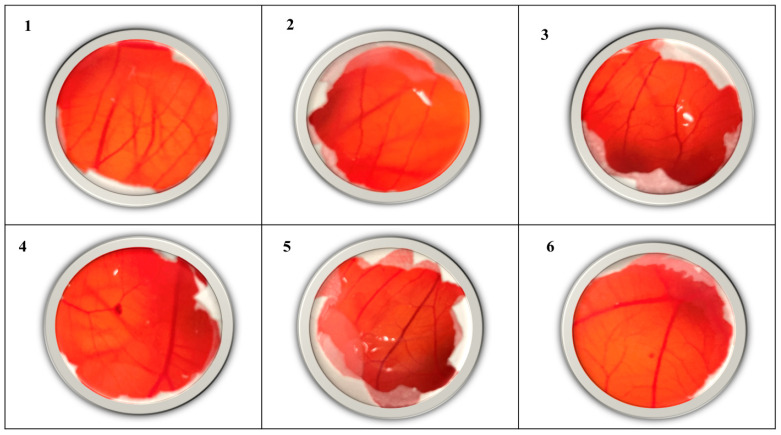
HET-CAM images of all groups (**1**: HPM; **2**: NE-10 Placebo; **3**: NE-HPM-10; **4**: CP1; **5**: CP2; **6**: CP:3).

In this research, the in vivo toxicity of all tested substances was evaluated using the HET-CAM assay with a sample size of *n* = 6 eggs per treatment group. The *H. perforatum* L. macerate (HPM) and the nanoemulsion formulation NE-HPM-10 induced observable reactions in the chorioallantoic membrane (CAM) within the 300s observation period. Conversely, the NE-HPM-10 placebo dispersion and the negative control elicited no discernible reactions in the CAM within the same timeframe. Among the three commercial preparations evaluated, hemorrhage and lysis were observed in the CAM of three out of six eggs within the initial 30 s. Beyond the 30 s mark, hemorrhage was noted in the CAM of the remaining eggs treated with commercial preparations. In the positive control group, at least two of the three endpoint reactions (lysis, hemorrhage, or coagulation) were observed within the first 30 s in each egg. Based on these observations, cumulative toxicity scores were assigned to each sample, and the mean score for each treatment group (*n* = 6 eggs) was calculated (Table 5). A summary of the HET-CAM assay results is presented in Table 5 and Figure 8. All HET-CAM toxicity tests were performed on *n* = 6 samples. In HPM and NE-HPM-10 dispersion, a reaction was observed in the CAM within 300 s. In the NE-HPM-10 dispersion of the placebo and the negative control, no reaction was observed in the CAM after 300 s. For all three commercial preparations utilized in the clinic, three of the six eggs exhibited both hemorrhage and lysis in the CAM within the initial 300 s. In the CAM, hemorrhage was observed after 300 s. In the positive control group, out of lysis, hemorrhage and coagulation reactions, at least two were observed within the first 30 s in each egg. According to these results, the scores indicated in Table 5 were assigned to each sample analyzed, and the mean of the six eggs tested for each sample was calculated. The results obtained are presented in Table 5 and Figure 8, respectively.

The findings of this study, which employed the HET-CAM test—a recent addition to the field of toxicology—demonstrated that both the HPM and NE-HPM-10 dispersions exhibited signs of irritation. Conversely, the NE-HPM-10 placebo dispersion and the negative control exhibited no such irritation (see Figure 8). The commercial preparations (CP1, CP2, and CP3) also demonstrated non-irritant profiles, despite the reactions observed in CAM. These findings provide valuable information regarding the in vivo toxicity of the substances tested, which may inform further development and safety considerations for potential wound-healing applications. Therefore, it also demonstrates the topical compatibility of NE-HPM-10.

### 2.11. In Vivo Clinical Findings

The results show that wound-healing scores differed significantly between the groups on days 4, 8, and 12 when compared to the control group (*p* < 0.05). The HPM group exhibited relatively low wound-healing scores (12th day/4.58 ± 0.58 mm). In contrast, the NG-2 group demonstrated markedly higher wound-healing scores (12th day/2.92 ± 0.38 mm). The NG-2 formulation, which is a nanoemulsion-based system, significantly accelerated wound closure. On both days 8 and 12, the scores were statistically higher than those of the control group. Both the CP1 (12th day/5.92 ± 1.16 mm) and CP2 (12th day/3.33 ± 0.41 mm) groups exhibited moderate wound-healing scores. These findings underscore the role of nanoemulsion-based delivery systems in enhancing wound-healing efficacy. The CP3 group (12th day/3.25 ± 0.52 mm) also showed high wound-healing scores and, together with NG-2, was identified as one of the most effective treatments. In the control group, wound-healing scores remained consistently low, and wound closure progressed at a slower rate compared to all treatment groups.

### 2.12. Clinical and Histopathological Findings

The histopathological evaluation compared the healing of connective and epithelial tissues in the wound area across different treatment groups. The degree of epithelialization and defect closure in the injured tissue was assessed.

**Figure 9:**
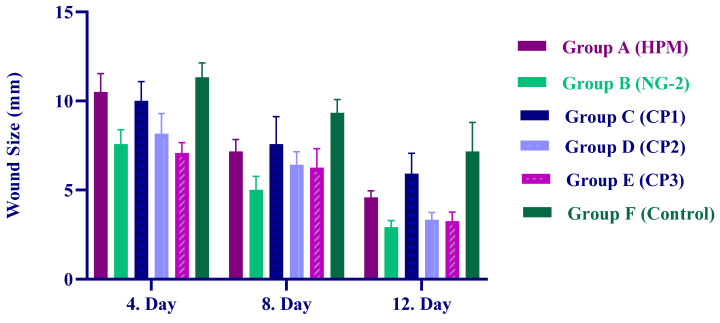
Wound-healing score of Group A (HPM), Group B (NG-2), Group C (CP1), Group D (CP2), Group E (CP3), and Group F (Control) in the skin defect area after 4, 8, and 12 days.

In the control group, which received no treatment and was allowed to heal naturally, the slowest healing rate was observed by the end of the study. This group exhibited minimal angiogenesis and collagen synthesis, and the wound margins remained unconnected, indicating incomplete healing (Figure 9).

**Figure 10:**
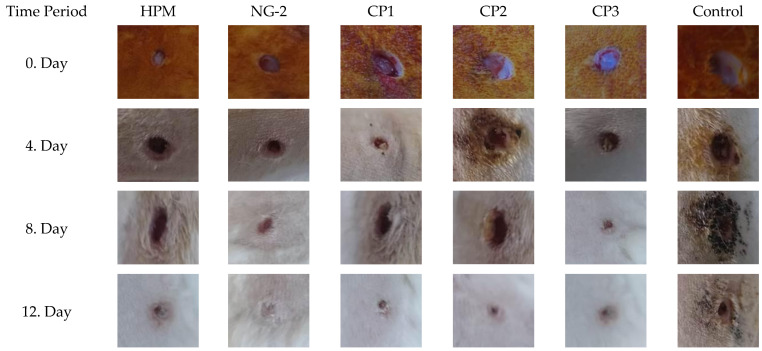
Wound-healing images of Group A (HPM), Group B (NG-2), Group C (CP1), Group D (CP2), Group E (CP3), and Group F (Control) in the skin defect area after 4, 8, and 12 days.

Similarly, in the group where only the hydrogel carrier component was applied, healing outcomes were comparable to those of the control group. Among the commercial preparations, CP3 demonstrated a more pronounced effect on wound healing compared to CP1. Notably, nanoemulsion-based formulations exhibited the most effective wound-healing outcomes (Figure 9 and Figure 10), suggesting their potential therapeutic advantage in promoting tissue regeneration.

**Figure 11:**
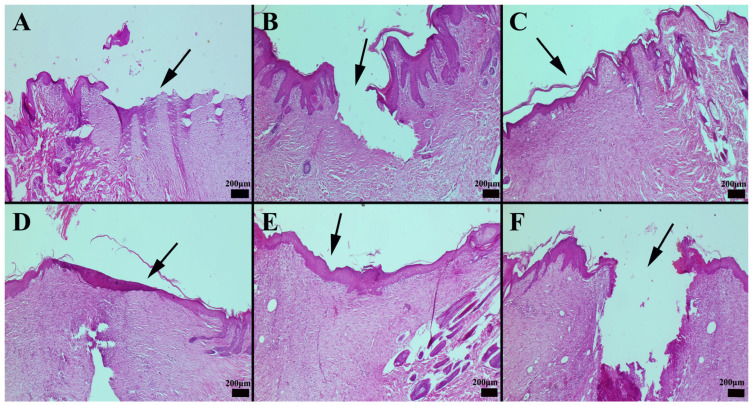
Histopathological appearance of wound healing in the skin defect area according to groups. (**A**) Group CP1 exhibited incomplete epithelialization and mild connective tissue proliferation; (**B**) group *Hypericum perforatum* macerate (HPM) showed initial epithelialization at the wound edges and increased connective tissue proliferation; (**C**) group nanoemulsion-*Hypericum perforatum* macerate + hydrogel (NG-2) displayed a significant increase in both epithelialization and connective tissue development; (**D**) group CP2 exhibited slight epithelial tissue closure and significant connective tissue healing; (**E**) group CP3 demonstrated modest yet notable epithelial tissue closure and substantial connective tissue healing; and (**F**) the control group showed minimal healing in both connective tissue and epithelialization, with the defect remaining patent. Arrows indicate the defect area (hematoxylin and eosin staining; scale bars = 200 µm).

Histopathological evaluation revealed that the commercial preparation CP2 facilitated the most pronounced wound-healing response, while the untreated control group exhibited the slowest rate of tissue regeneration (Figure 11). Notably, the CP2-treated group displayed elevated levels of angiogenesis and collagen deposition, indicative of accelerated tissue remodeling. Immunohistochemical analyses further demonstrated that CP2 treatment enhanced the expression of key growth factors, including vascular endothelial growth factor (VEGF), at the wound margins. Interestingly, nanoemulsion formulations elicited the highest levels of VEGF expression, with the specific composition of the nanoemulsion significantly influencing the observed outcome. Specifically, the NG-2 formulation was more effective in promoting growth factor expression than HPM alone.

### 2.13. Immunohistochemical Evaluation of Cytokeratin Expression

**Figure 12:**
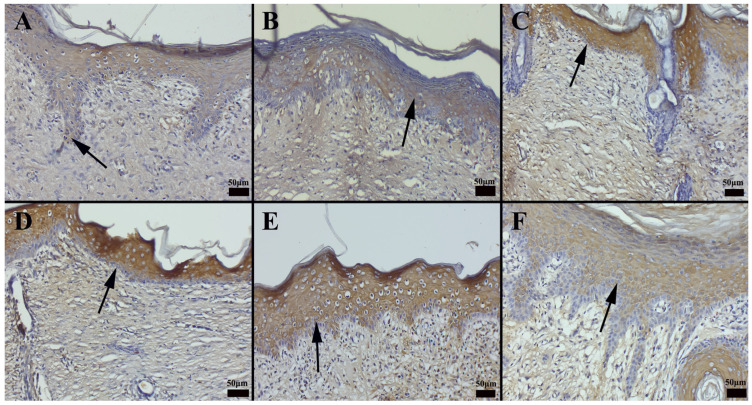
Immunohistochemical staining revealed the following patterns of cytokeratin expression across the treatment groups. (**A**) Group CP1 exhibited mild cytokeratin expression; (**B**) group *Hypericum perforatum* macerate (HPM) showed increased cytokeratin expression at the wound margins; (**C**) group nanoemulsion-*Hypericum perforatum* macerate + hydrogel (NG-2) displayed a significant increase in cytokeratin expression; (**D**) group CP2 exhibited mild cytokeratin expression in epithelial cells; (**E**) group CP3 demonstrated a moderate increase in cytokeratin expression; and (**F**) the control group showed minimal cytokeratin expression in only a few cells of the epithelial layer. Arrows indicate immunopositive cells (streptavidin-biotin peroxidase method; scale bars = 50 µm).

Immunohistochemical analysis of cytokeratin expression revealed significant intergroup differences. The CP3 group exhibited the most pronounced cytokeratin expression within newly epithelializing cells at the wound margins, while the control group showed the lowest levels of expression. Increased cytokeratin expression was also observed in the CP1-treated group and the group treated with the hydrogel carrier alone. Interestingly, treatment with olive oil was found to enhance cytokeratin expression during wound healing. Notably, the nanoemulsion formulation elicited the highest levels of cytokeratin expression among all treatment groups (Figure 12). When the immunohistochemical results of cytokeratin expression were compared between the groups, it was found that the CP3 group had the most pronounced expression in the cells undergoing new epithelialization at the edges of the defect area, whereas the control group had the least expression. Increased expression was seen in the group treated with CP1, as well as in the group to which the hydrogel carrier substance was applied. It was discovered that olive oil was effective at boosting the expression of cytokeratin during the healing of wounds. According to the preparations, the nanoemulsion form had the highest expression.

**Figure 13:**
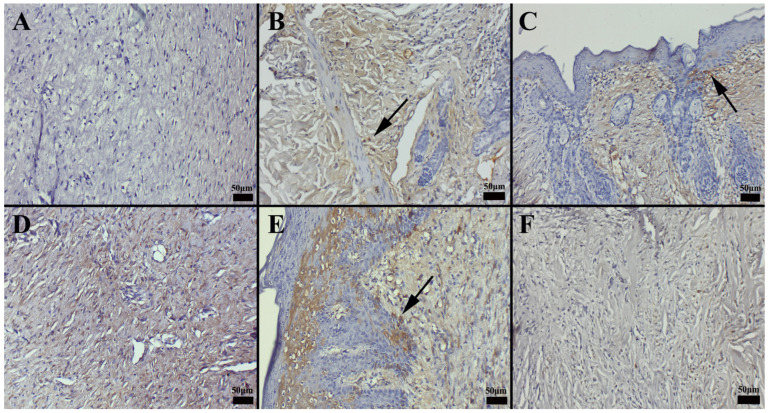
Immunohistochemical staining revealed the following patterns of VEGF expression across the treatment groups. (**A**) Group CP1 exhibited minimal VEGF expression; (**B**) group *Hypericum perforatum* macerate (HPM) showed a modest increase in VEGF expression, particularly within epithelial cells; (**C**) group nanoemulsion-*Hypericum perforatum* macerate + hydrogel (NG-2) displayed a significant increase in VEGF expression; (**D**) group CP2 exhibited mild VEGF expression in epithelial cells; (**E**) group CP3 demonstrated a moderate increase in VEGF expression within epithelial cells; and (**F**) the control group showed very faint VEGF expression in only a few cells. Arrows indicate immunopositive cells (streptavidin-biotin peroxidase method; scale bars = 50 µm).

Immunohistochemical analysis of vascular endothelial growth factor (VEGF) expression revealed varying levels of immunostaining in both epithelial and connective tissue cells across the treatment groups. The commercial preparation CP3 exhibited the most pronounced VEGF expression, while the control group showed minimal VEGF immunoreactivity. Notably, the nanoemulsion formulations elicited the highest levels of VEGF expression among all treatment groups, dependent on the specific formulation (Figure 13). A summary of the statistical analysis of immunohistochemical expression levels by treatment group is presented in Table 5.

Hypericum extracts have been shown to downregulate COX-2 expression, contributing to reduced inflammatory mediator release at the wound site. Furthermore, immunohistochemical and histochemical findings provide valuable information on cellular and extracellular matrix changes associated with the wound-healing process in different treatment groups. Nanoemulsion-based formulations consistently demonstrated superior results in terms of epithelialization and collagen deposition, thus providing evidence of their potential therapeutic advantage in promoting tissue regeneration (see Figure 11 and Figure 12).

**Figure 14:**
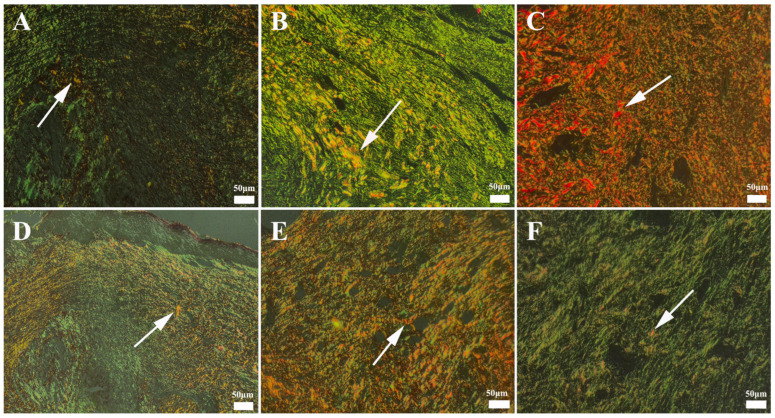
Appearance of Type III and Type I collagen formation according to groups. (**A**) Group CP1 showing very prominent Type III collagen (green) and very slight Type I collagen (red) (arrow), (**B**) group *Hypericum perforatum* macerate (H.P.M.) showing significant Type III collagen and slight Type I collagen (arrow), (**C**) group nanoemulsion form-*Hypericum perforatum* macerate + hydrogel (NG-2) showing a significant decrease in Type III and an increase in Type I collagen (arrow), (**D**) group CP2 showing a significant decrease in Type III and an increase in Type I collagen (arrow), (**E**) group CP3 showing decreased Type III and increased Type I collagen (arrow), and (**F**) control group showing very prominent Type III collagen and very slight Type I collagen (arrow). Arrows indicate areas with red fluorescence, representing mature Type I collagen. Picro-Sirius Red method, bars = 50 µm.

Picro-Sirius Red staining, employed to evaluate collagen deposition and organization within the connective tissue, revealed distinct patterns across the treatment groups. The defect areas exhibited a greater intensity of green fluorescence, indicative of newly synthesized, thin Type III collagen fibrils. Furthermore, varying degrees of increased red–orange fluorescence, corresponding to thicker, more mature Type I collagen fibers, were observed. The nanoemulsion-treated group exhibited the most substantial increase in Type I collagen deposition, while the control group displayed the lowest levels (Figure 14).

In an in vivo wound-healing model, the healing processes of different formulations were compared, with the Madecassol ointment group providing the best healing, followed by the CP3 and CP1 groups, while the control group showed the slowest healing. NG-2 was found to be more effective than olive oil, and *H. perforatum* macerate performed better in nanoemulsions, showing that it has a better potency in promoting wound healing (see Figure 14).

### 2.14. Statistical Analysis and Findings

The data are presented as the mean ± standard deviation (SD). Group differences were analyzed using one-way ANOVA, followed by the Duncan test for post hoc comparisons. Statistically significant differences between groups, indicated by different superscripts within the same column, were observed (*p* < 0.001).

The findings of this study suggest that nanoemulsion-based drug formulations significantly enhance wound healing. While the HPM alone showed limited effectiveness, formulations NG-2, CP3, and CP1 demonstrated notable improvements in the wound-healing process (Table 6).

The statistical findings presented in Table 6 provide further evidence to support the histopathological and immunohistochemical observations made previously. These findings serve to further strengthen the conclusion that nanoemulsion-based formulations demonstrate superior wound-healing potential in comparison to other treatment groups.

## 3. Conclusions

In this research paper, a nanoemulsion-based hydrogel formulation (NG-2) incorporating *Hypericum perforatum* L. macerate was successfully developed and comprehensively characterized for its potential in advanced wound-healing applications. The NG-2 formulation demonstrated favorable physicochemical attributes, including a nanoscale particle size, high encapsulation efficiency, and a stable zeta potential, which collectively contributed to improved drug delivery performance and controlled release kinetics, as confirmed by the Korsmeyer–Peppas model. The hydrogel matrix further enhanced the topical application properties by providing pseudoplastic, non-Newtonian flow behavior, optimal for sustained wound site adherence and prolonged drug release. In vitro assays revealed significant antioxidant and antimicrobial activities, particularly against methicillin-resistant *Staphylococcus aureus* (MRSA), underscoring the potential of nanoemulsification in amplifying the therapeutic efficacy of bioactive compounds derived from *H. perforatum* L. The non-irritant profile of the formulation, validated through HET-CAM testing, attests to its biocompatibility and clinical safety for topical use. In vivo wound-healing experiments revealed that NG-2 markedly accelerated re-epithelialization, enhanced collagen deposition, and stimulated angiogenesis, as evidenced by elevated VEGF and cytokeratin expression. Histopathological and immunohistochemical analyses confirmed the superior tissue regeneration capability of the NG-2 formulation compared to commercial benchmarks. These findings position NG-2 as a promising multifunctional platform for translational applications in wound care, offering a synergistic strategy that integrates the bioactivity of phytotherapeutics with the delivery advantages of nanoemulsion–hydrogel systems. Further research focusing on clinical evaluation and scale-up production is warranted to validate its applicability in dermal regenerative medicine and pharmaceutical formulations. Future studies will focus on further optimizing the formulation and conducting comprehensive clinical trials to validate its efficacy and safety in human subjects.

## 4. Materials and Methods

### 4.1. Plant Samples and Material Used

The *H. perforatum* L. plant samples used in this research were obtained from the provinces of Isparta and Antalya, Türkiye. To prepare the macerates, *H. perforatum* L. plants were collected from a mountainous region between the coordinates 37°47′56.6″ N, 30°50′22.9″ E and 37°42′32.0″ N, 30°50′52.7″ E, at an altitude of 1100–2000 m.

The *Hypericum perforatum* L. plant used in this research was collected from the Isparta province of Türkiye and deposited in the Isparta Süleyman Demirel University Gul Herbarium (Isparta, Türkiye) under the accession number GUL 27/1/67-1.

Nanoemulsions were prepared using the emulsification-solvent evaporation technique. All chemicals used in the formulation of hydrogels and antibacterial tests were of analytical grade and employed without any preliminary treatment. Ultrapure water was used in all synthesis and antibacterial activity experiments.

### 4.2. In Silico Study

A design of experiments (DoE) methodology was employed for formulation optimization, utilizing a D-optimal mixture design implemented in Design-Expert 13 software (Statease, Minneapolis, MN, USA). The ratios of oil and surfactant (Cremophor RH40) were defined as independent variables, while particle size, particle size distribution, and zeta potential were incorporated into the model as dependent variables. A quadratic calibration model was applied for data analysis [33].

### 4.3. Preparation of Nanoemulsion Formulations

Nanoemulsions incorporating *Hypericum perforatum* L. were prepared based on a modified version of the method reported by Chuacharoen et al. [34]. In this research, *H. perforatum* L. macerates constituted the dispersed phase, while ultrapure water (U.W.) served as the continuous phase. Cremophor RH40 (C-RH 40), a nonionic surfactant, was employed to stabilize the formulation [35,36].

Initially, the nonionic surfactant was dissolved in distilled water under agitation at 800 rpm for 30 min. Subsequently, the oil and water phases were combined and subjected to high-speed homogenization (Velp OV5 Ultra-Turrax, Usmate, Italy) at 12,000 rpm for 4 min to generate the core emulsion. Following confirmation of core emulsion formation, probe sonication (Bandelin, Berlin, Germany) was performed in an ice-water bath at 30% amplitude for durations of 1, 2, and 3 min to prevent overheating.

### 4.4. Preparation of Hydrogel Formulations

In the development of hydrogel formulations, Ultrez 21 and Ultrez 30 were utilized as the primary components. In the formulations, the amounts of Ultrez 21 and Ultrez 30 were standardized to be 0.5% [36]. To ensure a homogeneous dispersion of these polymers, they were hydrated by stirring in ultrapure water. Subsequently, the nanoemulsion containing standardized *H. perforatum* L. macerate was added to the system under continuous stirring conditions. The pH value of the formulations was then titrated to the range 6.5–7.5 using 0.1 M triethanolamine solution prepared with ultrapure water. Finally, the morphological and physicochemical properties of the obtained hydrogel formulations were studied in detail. All formulation ingredients are given in Table 7 and Table 8.

### 4.5. Characterization Studies of Nanoemulsion Formulations

#### 4.5.1. Dynamic Light Scattering (DLS) Analysis

The polydispersity index (PDI) and zeta potential of the NE-HPM nanoemulsions were determined at 25 ± 2 °C using a Zetasizer Nano ZSP (Malvern Instruments, Worcestershire, UK) employing dynamic light scattering (DLS) and electrophoretic mobility techniques. Before analysis, NE-HPM nanoemulsions (5 mL) were dispersed in 20 mL of deionized water. Measurements were performed with the refractive index set to 1.33 (for water at 25 °C) for the dispersing medium and 1.45 for liposome dispersions, serving as a reference standard. Subsequently, samples were diluted in ultrapure water until the count rate (expressed in kilo counts per second, kcps) decreased below 500. Following dilution, samples were analyzed in disposable plastic cuvettes. All measurements were performed in triplicate, with each trial consisting of 10 repeated acquisitions (*n* = 3).

#### 4.5.2. Morphological Characterization

The diameter, homogeneity, and morphological features of the nanoemulsion (NE) formulations were analyzed using scanning electron microscopy (SEM) (Zeiss EVO40, Oberkochen, Germany). For sample preparation, 5 μL of the nanoemulsion (2.5 mg/mL) was applied to a carbon film-coated copper grid (200 mesh) affixed with double-sided adhesive tape and allowed to air-dry for 2 min. To ensure optimal conductivity, the samples were sputter-coated with a thin conductive gold layer (10 nm thickness), adhering to established protocols [37]. SEM imaging was performed at an accelerating voltage of 10 keV under high-vacuum conditions, with secondary electron detection employed to capture high-resolution topographical details.

#### 4.5.3. Encapsulation Efficiency of Nanoemulsion

The encapsulation efficiency of hypericin in the nanoemulsion formulations was determined chromatographically, following the method described by Tatsis et al. [38]. The prepared dispersions were subjected to ultracentrifugation at 15,000 rpm for 30 min at 4 °C, resulting in the separation of the samples into a supernatant and a coagulated phase, as outlined by Akula et al. (2014) [39]. The amount of encapsulated hypericin was then quantified using high-performance liquid chromatography (HPLC) (Agilent 1100, Agilent, Santa Clara, CA, USA) at 270 nm, based on a predetermined regression equation (R^2^ = 0.9994, y = 157.717 + 189.4329). All analyses were performed in triplicate to ensure accuracy and reproducibility.

(1) $$\%EE=\frac{Total\;Amount\;of\;Hypericin-Amount\;of\;Hypericin\;in\;the\;Supernatantı}{Total\;Amount\;of\;Hypericin\;Substance}$$

#### 4.5.4. In Vitro Release Study

The in vitro release profile of the nano-hydrogel (NG-2) was evaluated using the dialysis bag method, with phosphate-buffered saline (PBS, pH 7.4) as the release medium. The hydrogel and nanoemulsion of the NG-2 formulation were based on previous characterization and in silico studies and were studied with 50 mg of sample. The hydrogel (NG-2), containing the active ingredient hypericin, was placed inside a dialysis membrane with a pore size of 2.4 nm, allowing the diffusion of molecules within the 3–5 kDa range. Before the experiment, the membrane was pre-soaked in ultrapure water for 30 min to ensure proper hydration.

The dialysis membrane was subsequently immersed in 30 mL of PBS (pH 7.4) and maintained at 37 ± 0.5 °C under continuous stirring. Samples (500 µL) were collected at predefined time points (0.5, 1, 2, 3, 4, 5, 6, 7, 8, 9, 10, 11, 12, 24, 36, 48, 60, 72, and 80 h) and analyzed via high-performance liquid chromatography (HPLC) following appropriate dilution, as described by Sarabandi et al. (2019) [40]. To maintain sink conditions and ensure accurate quantification, an equivalent volume (500 µL) of fresh pH 7.4 PBS was replenished after each sampling (*n* = 3). All experiments were conducted in triplicate (*n* = 3).

### 4.6. Rheological Characterization

The viscosity of NG1 and NG2 nanoemulsion hydrogels was assessed at 25 °C using a rheometer (Brookfield DV3T HA, Toronto, ON, Canada). Shear stress and viscosity values were recorded at progressively increasing shear rates. The rheological behavior of the formulations was analyzed by plotting viscosity and shear stress curves across different shear rate values, as outlined by Kolman et al. (2021) [41].

### 4.7. Stability Study

Stability assessments were conducted in accordance with the guidelines of the International Council for Harmonisation of Technical Requirements for Pharmaceuticals for Human Use (ICH). The formulations were stored under controlled conditions at 40 °C ± 2 °C/75% ± 5% RH, 25 °C ± 2 °C/60% ± 5% RH, and 5 °C ± 2 °C within stability cabinets. Evaluations were performed at predetermined intervals, specifically on Day 0, Day 14, and at 1, 2, 3, and 4 months.

The stability of the nanoemulsion hydrogel formulations was assessed by monitoring changes in particle size and zeta potential over time. Additionally, physicochemical properties, visual appearance, pH variations, encapsulation efficiency, and viscosity were analyzed to determine the overall stability of the formulations, as described by González-González et al. (2022) [42].

### 4.8. In Vitro Study

#### 4.8.1. In Vitro Antimicrobial Activity

The in vitro antimicrobial activities of the nanoemulsion formulations (NFs) were evaluated using the microdilution method [43,44]. Antimicrobial activity was assessed against a panel of bacterial strains, including Gram-negative bacteria *Escherichia coli* (ATCC 25922) and *Pseudomonas aeruginosa* (ATCC 27853); Gram-positive bacteria *Bacillus cereus* (ATCC 11778), *Enterococcus faecalis* (ATCC 29212), *Staphylococcus aureus* (ATCC 29213), and methicillin-resistant *Staphylococcus aureus* (MRSA) (ATCC 43300); and the fungal pathogen *Candida albicans* (ATCC 90028).

All microorganism strains were incubated for 24 h. The microdilution assay was performed using 96-well microplates and Mueller Hinton Broth (MHB; Merck, Darmstadt, Germany) as the growth medium. Nanoemulsion formulations were prepared at predetermined concentrations (100 µg formulation/200 µL organic solvent) and serially diluted within the microplate wells. Following the dissolution of the nanoemulsions in the solubilizer, they were added to the growth medium.

Microorganism suspensions were adjusted to a 0.5 McFarland standard, followed by the addition of 5 µL of each suspension to the microplate wells. Microplates were incubated at 37 °C for 24 h. The minimum inhibitory concentration (MIC), defined as the lowest concentration at which visible growth was inhibited, was then recorded [40]. To determine the minimum bactericidal concentration (MBC), 100 µL aliquots from the microplate wells were transferred to Mueller Hinton Agar (MHA) medium and incubated at 37 °C for 24 h.

Physiological saline (S.F.) served as the negative control, while oxytetracycline (CP3) and a commercial formulation (CP1) were used as positive controls. All tests and controls were performed in triplicate to ensure data reliability.

#### 4.8.2. In Vitro Antioxidant Activity

##### Determination of DPPH (2,2-Diphenyl-1-Picrylhydrazyl) Radical Scavenging Activity

The antioxidant activity of the nanoparticle (NP) formulations was evaluated using the DPPH radical scavenging assay. NPs were prepared at a concentration of 1 mg/mL in methanol, and a 200 mmol/L DPPH solution was added to the mixture. The samples were then incubated for 30 min, during which a color change was observed, indicating the reduction in DPPH radicals.

Following incubation, the absorbance of the samples was measured at 517 nm. The ability to scavenge DPPH radicals was calculated using the following equation [43,44]:

(2) $$DPPH\;radical\;scavenging\;effect\;(\%)=\frac{Control\;absorbance-Sample\;absorbance}{Control\;absorbance\;\times \;100}$$

##### ABTS Radical Scavenging Assay

A stock solution of 2,2′-azino-bis(3-ethylbenzothiazoline-6-sulfonic acid) diammonium salt (ABTS) was prepared by dissolving 383 mg of ABTS (7 mM) and 66.2 mg of potassium persulfate (2.45 mM) in 100 mL of methanol. The mixture was briefly vortexed and then allowed to stand in complete darkness for 5 min to facilitate complete radical formation. The absorbance was subsequently measured at 734 nm. The ABTS radical scavenging activity, expressed as the IC50 value, was determined by quantifying the concentration of hypericin required to inhibit 50% of the ABTS radicals.

##### Ferric Reducing Antioxidant Power (FRAP) Assay

The FRAP reagent was pre-warmed to 37 °C. Subsequently, a 150 µL aliquot of the sample was added to 2.85 mL of the warmed FRAP reagent in a cuvette, and the reaction mixture was incubated in the dark for 30 min. The formation of the colored ferrous tripyridyl triazine complex was then quantified spectrophotometrically by measuring the absorbance at 593 nm. A linear calibration curve was generated using Trolox standards ranging from 25 to 800 µM. FRAP values were expressed as millimoles of Trolox equivalents (TE) per gram of sample [45,46,47,48].

#### 4.8.3. Hen’s Egg Test–Chorioallantoic Membrane (HET-CAM) Assay

The in ovo toxicity assessment was conducted following the Interagency Coordinating Committee on the Validation of Alternative Methods (ICCVAM) guidelines [49,50] employing the Hen’s egg test–chorioallantoic membrane (HET-CAM) assay as an alternative to the Draize test. Fertile White Leghorn hen eggs, weighing 50–60 g, were utilized as the experimental substrate. The eggs were incubated at 37.8 ± 0.3 °C and 58 ± 2% relative humidity for nine days in a forced-air incubator equipped with an automated rotation mechanism. On day nine, an aperture was carefully created in the eggshell using a rotary tool to expose the chorioallantoic membrane (CAM).

For the toxicity assessment, *H. perforatum* L. macerate and the NE-HPM-10 formulation were prepared at a concentration of 6.5 mg/mL in the experimental groups. The commercial preparations were homogenized to a concentration of 1 g/mL by diluting 10 g samples with 10 mL of phosphate-buffered saline (PBS) at 10,000 rpm. Subsequently, 300 µL of the test solutions was applied topically to the vascularized CAM. Sodium hydroxide solution (0.1 N) and isotonic 0.9% NaCl solution served as the positive and negative controls, respectively. CAM reactions were monitored for 300 s, and the time of onset for each endpoint (lysis, hemorrhage, or coagulation) was recorded [51]. A cumulative toxicity score was calculated based on the time of endpoint occurrence (Table 9). Individual scores were generated for each sample within a test group, and the mean score for the group was calculated. The overall toxicity of each formulation was then evaluated based on the corresponding toxicity score (Table 10).

### 4.9. In Vivo Wound-Healing Study

#### 4.9.1. In Vivo Debridement Wound Study

The present investigation was conducted under the auspices of the Burdur Mehmet Akif Ersoy University Experimental Animals Ethics Committee approval. Male New Zealand White rabbits, weighing between 1000 and 1500 g and aged 30 to 38 weeks, were utilized in this research. A debridement wound model was employed, and a total of 36 wounds were surgically created and randomly assigned to six treatment groups (*n* = 6 wounds/group): Group A (HPM), Group B (NG-2), Group C (CP1), Group D (CP2), Group E (CP3), and Group F (Control). Treatment regimens were administered topically four times daily for 12 days to all groups, except the untreated control group.

#### 4.9.2. Clinical Evaluation

In this research, the progression of wound closure in rabbits was monitored via calliper measurements taken on postoperative days 4, 8, and 12. Concurrent with these measurements, a comprehensive clinical evaluation of each wound site was performed. Regarding the clinical findings of our study; the wounds in the rabbits in the postoperative period were measured with the help of calipers at the same time on the 4th, 8th, and 12th days and the sizes of the measured wounds are shown in Figure 9, according to the 6 different groups [52].

The experimental groups were as follows:

- Group A: HPM (*H*. *perforatum* macerate applied to the wound);
- Group B: NG-2 (*H*. *perforatum* nanoemulsion in hydrogel applied to the wound);
- Group C: CP1 (Commercial product 1 applied to the wound);
- Group D: CP2 (Commercial product 2 applied to the wound);
- Group E: CP3 (Commercial product 3 applied to the wound);
- Group F: Control (Isotonic 0.9% NaCI applied to the wound).

#### 4.9.3. Histopathological Examination

Post-wound healing skin samples from rabbits were harvested after day 12 for histopathological analysis and fixed in 10% buffered formaldehyde. The trimmed tissue samples were processed using standard histological procedures with a tissue processor (Leica ASP300S, Leica Biosystems, Nürnberg, Germany) and subsequently embedded in paraffin blocks. Serial sections, each 5 µm thick, were obtained using a Leica 2155 rotary microtome and mounted onto glass slides.

The tissue sections were deparaffinized and rehydrated following overnight drying. Hematoxylin and eosin (H&E) staining was performed on one set of sections for routine histopathological analysis and light microscopy evaluation. To assess connective tissue repair, an additional set of sections was stained using the Picro-Sirius technique [53]. For this purpose, sections from the defect area were deparaffinized, rehydrated, and stained with Picro-Sirius Red using a commercially available kit (ab150681, Abcam, UK), following the manufacturer’s instructions.

#### 4.9.4. Immunohistochemical Analysis

For immunohistochemical analysis, two series of sections were prepared during microtomy and mounted onto poly-L-lysine-coated slides. The sections were then immunostained for vascular endothelial growth factor (VEGF) [VEGF (C-1) antibody, sc-7269 Santa Cruz, Dallas, TX, USA, 1:100 dilution] and cytokeratin-1 [cytokeratin 1 (4D12B3): sc-65999, 1:100 dilution] following the manufacturer’s protocol for the streptavidin-biotin complex peroxidase method [53,54]. A Mouse and Rabbit Specific HRP/DAB IHC Detection Kit–Micro-polymer (ab236466, Abcam, Cambridge, UK) was employed as the secondary antibody, with diaminobenzidine (DAB) serving as the chromogen. Negative control sections were incubated with an antibody dilution buffer in lieu of the primary antibody. A board-certified pathologist, blinded to the experimental groups and affiliated with an external institution, performed all assessments. Immunohistochemical expression was graded using a semi-quantitative scoring system: 0 (negative), 1 (mild), 2 (moderate), and 3 (strongly positive). Statistical analysis was conducted on the resulting scores to determine intergroup differences. Microphotographs were acquired and morphometric analyses performed using the CellSens Life Science Imaging Software system (Olympus Corporation, Tokyo, Japan), and immunohistochemical results were quantified using ImageJ software (version 1.48, National Institutes of Health, Bethesda, MD, USA).

### 4.10. Statistical Analysis

Statistical analyses of histopathological scores and the number of immunohistochemically positive cells were conducted using SPSS 22.0 software. The Shapiro–Wilk test was initially performed to evaluate the normality of data distribution. As the data followed a normal distribution (*p* > 0.05), one-way analysis of variance (ANOVA) was applied to compare the groups. Pairwise comparisons were conducted using the post hoc Duncan test to identify significant differences between groups. A significance level of *p* < 0.05 was considered statistically significant, and results are expressed as the mean ± standard deviation (SD).

## Figures and Tables

**Table 1:** Ingredients and codes of nanoemulsion formulations.

Formulation Code	Ingredients	Con. Ratio (%)
NE-HPM-1	HPM:C-RH40:U.W.	9.0:7.6:83.4
NE-HPM-2	HPM:C-RH40:U.W.	7.7:6.4:85.9
NE-HPM-3	HPM:C-RH40:U.W.	7.7:7.4:84.9
NE-HPM-4	HPM:C-RH40:U.W.	9.0:6.4:84.6
NE-HPM-5	HPM:C-RH40:U.W.	7.0:8.0:85.0
NE-HPM-6	HPM:C-RH40:U.W.	7.7:5.4:86.9
NE-HPM-7	HPM:C-RH40:U.W.	6.0:6.9:87.1
NE-HPM-8	HPM:C-RH40:U.W.	6.0:5.9:88.1
NE-HPM-9	HPM:C-RH40:U.W.	9.0:5.1:85.9
NE-HPM-10	HPM:C-RH40:U.W.	6.5:5.0:88.5
NE-HPM-11	HPM:C-RH40:U.W.	7.8:5.0:87.2

NE: Nanoemulsion, HPM: *H. perforatum* L. macerate, C-RH40: Cremophor RH40: U.W.: ultrapure water.

**Table 2:** Characteristics of HPM-loaded nanoemulsions in terms of mean particle size, PDI, zeta potential, and encapsulation efficiency (*n* = 3).

Formulation Code	Batch Composition (*w*/*w*/*w*/*w*)	Particle Size (nm) ± SD	Polydispersity Index (PDI) ± SD	Zeta Potential (mV) ± SD	Encapsulation Efficiency (%) ± SD
NE-HPM-1	HPM:C-RH40:U.W. (9.0:7.6:83.4)	188.9 ± 5.373	0.347 ± 0.039	−17.4 ± 0.7	97.77 ± 7.21
NE-HPM-2	HPM:C-RH40:U.W. (7.7:6.4:85.9)	189.8 ± 4.508	0.310 ± 0.013	−14.0 ± 0.2	96.78 ± 6.34
NE-HPM-3	HPM:C-RH40:U.W. (7.7:7.4:84.9)	193.8 ± 5.707	0.364 ± 0.008	−18.1 ± 0.4	98.45 ± 7.65
NE-HPM-4	HPM:C-RH40:U.W. (9.0:6.4:84.6)	191.1 ± 6.366	0.325 ± 0.049	−20.7 ± 1.0	97.63 ± 7.09
NE-HPM-5	HPM:C-RH40:U.W. (7.0:8.0:85.0)	190.7 ± 2.635	0.317 ± 0.028	−18.0 ± 0.2	95.81 ± 6.27
NE-HPM-6	HPM:C-RH40:U.W. (7.7:5.4:86.9)	184.1 ± 4.255	0.358 ± 0.014	−22. 9± 0.1	93.56 ± 6.09
NE-HPM-7	HPM:C-RH40:U.W. (6.0:6.9:87.1)	185.0 ± 4.779	0.315 ± 0.031	−19.8 ± 0.7	96.31 ± 4.679
NE-HPM-8	HPM:C-RH40:U.W. (6.0:5.9:88.1)	176.9 ± 4.355	0.348 ± 0.050	−18.3 ± 0.5	89.72 ± 6.19
NE-HPM-9	HPM:C-RH40:U.W. (9.0:5.1:85.9)	179.6 ± 1.750	0.326± 0.050	−17.9 ± 0.3	94.33 ± 4.91
NE-HPM-10	HPM:C-RH40:U.W. (6.5:5.0:88.5)	174.8 ± 1.436	0.274± 0.023	−23.3 ± 0.2	99.83 ± 3.09
NE-HPM-11	HPM:C-RH40 U.W. (7.8:5.0:87.2)	176.8 ± 2.007	0.324 ± 0.054	−18.6 ± 0.8	97.27 ± 5.16

**Table 3:** Kinetic models of NG-2 (as hypericin).

Kinetic Model		Zero Order	First Order	Higuchi	Hixson–Crowell	Korsmeyer–Peppas
NG-2	Slope	0.8888	−0.0065	1.7481	0.0191	1.2067
	Intercept	−2.7896	2.0332	−0.7975	−0.0855	−0.449
	R	0.978	0.942	0.964	0.956	0.980
	r^2^	0.9572	0.8867	0.930	0.9146	0.9606

**Table 4:** Stability study results of NG-2 nanoemulsion hydrogel formulation (*n* = 3).

Formulation	Time Period	Temperature (°C)	Size (d.nm)	Zeta (mV)	PDI	EE%	Viscosity (mPa.s)	pH
NG-2 Formulation	Day 0	-	174.800 ± 1.436	−23.3 ± 0.2	0.274 ± 0.023	99.83 ± 3.09	46,200 ± 070	7.322 ± 0.04
	Day 14	4 ± 2	174.932 ± 1.512	−23.2 ± 0.80	0.283 ± 0.032	98.93 ± 1.95	46,000 ± 125	7.128 ± 0.039
		25 ± 2	176.441 ± 2.302	−22.8 ± 1.5	0.299 ± 0.060	98.46 ± 1.01	46,200 ± 190	7.224 ± 0.026
		40 ± 2	180.093 ± 2.125	−20.8 ± 2.4	0.260 ± 0.100	93.95 ± 1.12	46,400 ± 235	7.196 ± 0.078
	1st month	4 ± 2	176.842 ± 1.064	−23.1 ± 1.1	0.290 ± 0.040	98.41 ± 1.03	45,930 ± 155	7.178 ± 0.031
		25 ± 2	179.144 ± 2.735	−22.9 ± 1.9	0.302 ± 0.020	98.04 ± 1.28	46,440 ± 130	7.211 ± 0.022
		40 ± 2	188.201 ± 2.924	−20.2 ± 2.1	0.321 ± 0.050	92.74 ± 1.84	46,540 ± 185	7.235 ± 0.056
	2nd month	4 ± 2	179.687 ± 1.302	−22.1 ± 1.2	0.291 ± 0.040	97.93 ± 1.22	45,710 ± 160	7.308 ± 0.039
		25 ± 2	183.605 ± 2.908	−21.7 ± 2.2	0.308 ± 0.030	97.58 ± 0.98	46,520 ± 140	7.202 ± 0.026
		40 ± 2	193.881 ± 2.507	−20.1 ± 2.3	0.334 ± 0.030	88.90 ± 1.03	46,800 ± 175	7.196 ± 0.078
	3rd month	4 ± 2	182.254 ± 1.401	−22.4 ± 1.4	0.296 ± 0.040	96.40 ± 1.03	45,680 ± 200	7.015 ± 0.031
		25 ± 2	191.857 ± 2.707	−21.2 ± 2.5	0.328 ± 0.020	92.20 ± 1.18	46,670 ± 180	7.203 ± 0.022
		40 ± 2	222.002 ± 2.663	−20.6 ± 2.4	0.354 ± 0.050	81.50 ± 1.19	46,700 ± 250	7.106 ± 0.056
	4th month	4 ± 2	184.284 ± 1.502	−21.7 ± 1.9	0.299 ± 0.070	92.70 ± 0.72	45,600 ± 210	6.803 ± 0.108
		25 ± 2	221.465 ± 2.904	−20.9 ± 2.7	0.334 ± 0.050	86.90 ± 1.35	46,730 ± 195	6.115 ± 0.089
		40 ± 2	267.028 ± 2.708	−20.01 ± 2.5	0.365 ± 0.070	73.70 ± 0.89	46,900 ± 290	6.012 ± 0.103

**Table 5:** Mean quandary and irritation evaluation in the HET-CAM test of HPM (6.5 mg/mL), NE-HPM-10 placebo, NE-HPM-10 (6.5 mg/mL), and placebos of all formulations administered: CP1, CP2, and CP3 (*n* = 6).

Formulation	Cumulative Score	Irritation Assessment
HPM	0.33	non-irritant
NE-10 placebo	0	non-irritant
NE-HPM-10	0	non-irritant
CP1	0.67	non-irritant
CP2	0.33	non-irritant
CP3	0.5	non-irritant

**Table 6:** Statistical analysis results of immunohistochemical scores according to the groups.

Group	Cytokeratin	VEGF
HPM	1.66 ± 0.51 ^a^	1.33 ± 0.51 ^ab^
NG-2	2.66 ± 0.51 ^b^	2.66 ± 0.51 ^c^
CP1	1.33 ± 0.51 ^a^	0.83 ± 0.40 ^a^
CP2	2.50 ± 0.54 ^b^	1.33 ± 0.51 ^ab^
CP3	2.50 ± 0.54 ^b^	2.50 ± 0.54 ^c^
Control	1.00 ± 0.53 ^a^	0.50 ± 0.22 ^a^

The data was presented as mean ± standard deviation. Differences between groups were analyzed using One-way ANOVA with Duncan test. Significant differences between groups with different superscripts in the same column were observed (*p* < 0.001).

**Table 7:** Hydrogel formulation components.

Ingredients	Concentration Ratio (%)
HPM-containing nanoemulsion	10
Propylene glycol	10
Glycerin	2
Gelling agents—U21 and U30	0.5
Triethanolamine (TEA)	0.5
Antimicrobial preservative	0.90
Ultrapure water	76.1

**Table 8:** Nanoemulsion-gel formulation codes and ingredients.

Formulation Code	Ingredients
NG1	U21 (%0.5) Gel + NE-HPM-10
NG2	U30 (%0.5) Gel + NE-HPM-10

**Table 9:** HET-CAM test scoring scheme for membrane irritation endpoints.

Effect	Score		
	30 s	120 s	300 s
Lysis	5	3	1
Bleeding	7	5	3
Coagulation	9	7	5

**Table 10:** Classification scheme for cumulative scores on the HET-CAM test.

Cumulative Score	Irritation Assessment
<0.9	Not toxic
1–4.9	Low toxicity
5–8.9	Moderately toxic
>9	High toxicity

## Data Availability

The original contributions presented in this study are included in the article. Further inquiries can be directed to the corresponding author.

## Abbreviations

The following abbreviations are used in this manuscript:

H.P.	*Hypericum perforatum* L.
HPM	*Hypericum perforatum* L. macerate
NG	Nanoemulsion hydrogel
NE-HPM	*Hypericum perforatum* L. nanoemulsion
C-RH 40	Cremophor RH40
U21	Ultrez 21
U30	Ultrez 30
DoE	Design of experiments
PDI	Polydispersity index
ICH	International Council for Harmonisation
NF	Nanoemulsion formulations
MRSA	Methicillin-resistant *Staphylococcus aureus*
MBC	Minimum bactericidal concentration
MIC	Minimum inhibitory concentration
CP	Commercial formulation
*FRAP*	Ferric reducing antioxidant power
CAM	Chorioallantoic membrane
HET-CAM	Hen’s egg test–chorioallantoic membrane
ICCVAM	Interagency Coordinating Committee on the Validation of Alternative Methods
VEGF	Vascular endothelial growth factor
